# The MUC5B Promoter Polymorphism is Not Associated With Non-ILD Chronic Respiratory Diseases or Post-transplant Outcome

**DOI:** 10.3389/ti.2022.10159

**Published:** 2022-05-16

**Authors:** Tinne Goos, Stijn E. Verleden, Laurens J. De Sadeleer, Anke Van Herck, Annelore Sacreas, Arno Vanstapel, Janne Kaes, Vincent Geudens, Celine Aelbrecht, David Ruttens, Diether Lambrechts, Sascha Vermeer, Laurens J. Ceulemans, Dirk E. Van Raemdonck, Laurent Godinas, Jonas Yserbyt, Bart M. Vanaudenaerde, Geert M. Verleden, Robin Vos, Wim A. Wuyts

**Affiliations:** ^1^ BREATHE, Department of Chronic Diseases and Metabolism, KU Leuven, Leuven, Belgium; ^2^ Department of ASTARC, University of Antwerp, Edegem, Belgium; ^3^ Department of Respiratory Medicine, University Hospital Antwerp, Edegem, Belgium; ^4^ Department of Thoracic and Vascular Surgery, University Hospital Antwerp, Edegem, Belgium; ^5^ Department of Pulmonary Medicine, Ziekenhuis Oost Limburg, Genk, Belgium; ^6^ Department of Medicine and Life Sciences, Hasselt University, Diepenbeek, Belgium; ^7^ Center for Cancer Biology, VIB, Leuven, Belgium; ^8^ Laboratory of Translational Genetics, Department of Human Genetics, KU Leuven, Leuven, Belgium; ^9^ Center for Human Genetics, University Hospitals Leuven, Leuven, Belgium; ^10^ Department of Thoracic Surgery, University Hospitals Leuven, Leuven, Belgium; ^11^ Department of Respiratory Medicine, University Hospitals Leuven, Leuven, Belgium

**Keywords:** lung transplantation, MUC5B, genetics, interstitial lung diseases, respiratory diseases

## Abstract

The MUC5B promoter polymorphism (rs35705950) has been associated with interstitial lung disease (ILD) and with prolonged pre-transplant survival in idiopathic pulmonary fibrosis (IPF), but no information is available regarding its prevalence in other respiratory diseases and its influence on post-transplant outcome. We included the Leuven lung transplantation cohort between 1991 and 2015 (*n* = 801). We assessed the minor allele frequency (MAF) of the MUC5B variant in the entire study cohort and investigated the influence of recipient MUC5B promoter polymorphism on post-transplant outcome in patients who were transplanted after 2004. MUC5B was successfully genotyped in 746 patients. The MAF was significantly higher in ILD (17.6%) compared to chronic obstructive pulmonary disease (COPD)/emphysema (9.3%), cystic fibrosis (CF)/bronchiectasis (BRECT) (7.5%) and pulmonary hypertension (PHT) (7.4%) (*p* < 0.001). No association was observed between rs35705950 and chronic lung allograft dysfunction (CLAD)/graft loss in the ILD population [CLAD: HR 1.37 95% CI (0.70–2.68); graft loss: HR 1.02 95% CI (0.55–1.89)], nor the entire study cohort [CLAD: HR 0.96 95% CI (0.69–1.34); graft loss: HR 0.97 95% CI (0.70-1.35)]. The MUC5B promoter polymorphism is a very specific predictive factor for the presence of pulmonary fibrosis as it is only associated with pulmonary fibrosis and not with other chronic respiratory diseases. While the MUC5B promoter variant is associated with better pre-transplant survival among IPF patients, recipient MUC5B promoter variant does not play a role in post-transplant outcome.

## Graphical Abstract

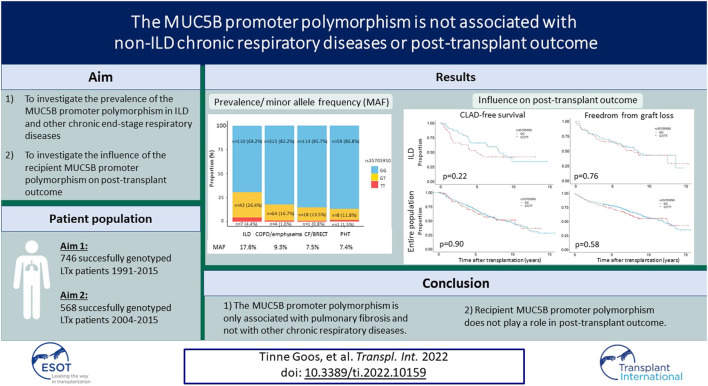

## Introduction

Family clustering of pulmonary fibrosis first suggested important roles for genomics in the underlying pathophysiology. In the last decade, several studies have identified rare and common genetic variants that are associated with pulmonary fibrosis. In 2011, Seibold et al. identified a common variant (rs35705950) in the promoter region of the mucin 5b (MUC5B) gene, which was associated with familial pulmonary fibrosis, sporadic idiopathic pulmonary fibrosis (IPF) and increased expression of mucin 5B in the lung (1). This identification suggested a potential role for the distal airways and mucus overproduction in the pathogenesis of pulmonary fibrosis. Since 2011, a significantly higher frequency of the minor T allele of the MUC5B promoter polymorphism has also been demonstrated in patients with idiopathic non-specific interstitial pneumonia (iNSIP), rheumatoid arthritis associated-interstitial lung disease (RA-ILD), chronic hypersensitivity pneumonitis (cHP), asbestosis and interstitial lung abnormalities, but not in patients with systemic sclerosis associated-ILD (SSc-ILD), myositis-associated ILD, antisynthetase syndrome and sarcoidosis (1–9). The prevalence of the minor T allele in other non-ILD chronic respiratory diseases such as chronic obstructive pulmonary disease (COPD)/emphysema, cystic fibrosis (CF)/bronchiectasis (BRECT) and pulmonary hypertension (PHT) is still unknown.

Although the MUC5B minor T allele and its increased expression of mucin 5B in the lung has been associated with IPF, IPF patients carrying the minor allele have reduced pre-transplant mortality compared to IPF patients without the minor allele (10). In contrast, rare pathogenic variants in telomere-related genes (e.g., TERT, TERC, PARN, and RTEL1) have been associated with increased mortality and poor post-transplant outcome (2,11–14). The influence of the recipient MUC5B polymorphism on post-transplant outcome is still unknown.

In this study, we assessed the prevalence of the MUC5B minor T allele in patients with ILD and according to ILD subtype, COPD/emphysema, CF/BRECT and PHT who underwent lung transplantation (LTx) at our center between 1991 and 2015. We compared the prevalence of the MUC5B promoter polymorphism between ILD and other chronic end-stage respiratory diseases. Furthermore, we investigated the influence of recipient MUC5B polymorphism on post-transplant incidence of chronic lung allograft dysfunction (CLAD) and graft loss in the ILD population and the entire population of patients who underwent lung transplantation for a chronic end-stage respiratory disease between 2004 and 2015.

## Methods

### Study Cohort

**FIGURE 1:**
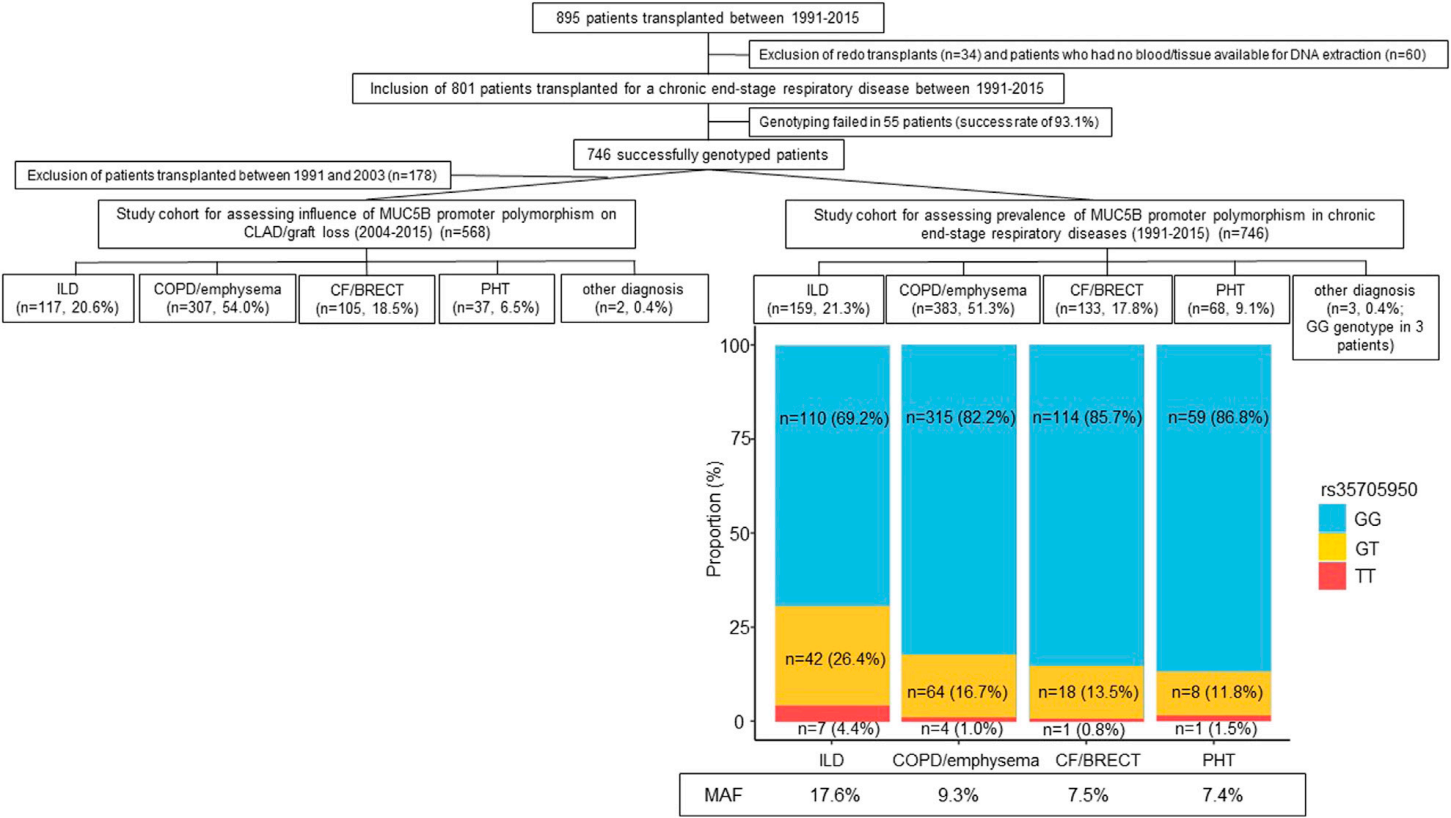
Flowchart of the study cohort with the prevalence and minor allele frequency of the MUC5B promoter polymorphism in chronic end-stage respiratory diseases. Abbreviations: CF, cystic fibrosis; BRECT, bronchiectasis; COPD, chronic obstructive pulmonary disease; ILD, interstitial lung disease; PHT, pulmonary hypertension; MAF, minor allele frequency.

Between 1991 and 2015, 895 patients were transplanted at the University Hospitals Leuven. Redo transplants (*n* = 34) and patients who had no blood or tissue available for DNA extraction were excluded (*n* = 60). The study cohort therefore encompassed 801 patients who were transplanted for a chronic end-stage respiratory disease between 1991 and 2015. Genotyping of MUC5B polymorphism (rs35705950) was performed in this cohort. Genotyping failed in 55 patients (success rate of 93.1%). The prevalence of the MUC5B promoter polymorphism was therefore assessed in 746 successfully genotyped patients: 159 patients had any form of ILD, 383 COPD/emphysema, 133 CF/BRECT, 68 PHT and 3 another diagnosis. As since 2004 all patients are uniformly treated with azithromycin and more electronic clinical data are available, the influence of recipient MUC5B polymorphism on post-transplant incidence of CLAD and graft loss has only been studied in patients who were transplanted after 2004. Therefore, for this analysis, 568 patients transplanted between 2004 and 2015 were included: 117 patients had any form of ILD, 307 COPD/emphysema, 105 CF/BRECT, 37 PHT and 2 another diagnosis. Study design is presented in Figure 1. Clinical information was retrospectively extracted from the electronic medical records. Patient follow-up was recorded until the October 17, 2019, resulting in a minimal follow-up of at least 4 years post-transplantation. This study was approved by our local Ethics Committee and all patients gave written informed consent to access their clinical and biobank data for research (S51577/S54739/ML5629).

### Genotyping

Recipient DNA was extracted from peripheral blood or from tissue of explanted lungs when no blood was available. A part of the samples has been previously used by Ruttens et al. (15). DNA from blood samples was extracted using the QIAamp DNA Blood Midi kit (Qiagen, Hilden, Germany) and from lung tissue by using Qiagen DNeasy Blood & Tissuekit. The Nanodrop-1000 (NanoDrop Technologies, Wilmington, DE, United States) was used for the control of DNA purity according to standard guidelines (260/280 ratio ∼1.7–1.9 and 260/230 ratio ∼2.0–2.2). DNA (5 ng/μL) was aliquoted into 384-well plates and genotyped at the Vesalius Research Center (Leuven).

Genotyping for MUC5B polymorphism (rs35705950) was performed using iPLEX technology on a MassARRAY Compact Analyzer (Sequenom Inc., San Diego, CA, United States) (16). This method is based on distinguishing allele-specific primer extension products by mass spectrometry (matrix-assisted laser desorption/ionization time-of-flight - MALDI-TOF). The MassARRAY RTTM software was used for generating automated genotyping calls followed by validation through manual review of the raw mass spectra. Results were paralleled with clinical patient data.

### Patient Characteristics and Outcome

Patient characteristics included age at time of LTx, gender, date of LTx, type of LTx [single lung transplantation (SL), sequential single lung transplantation (SSL) or heart-lung transplantation (HL)] and underlying disease. Clinical follow-up data were collected for ILD patients and included ischemic time, immunosuppressive treatment at time of discharge from intensive care unit, corticosteroid use and dose at time of pre-transplant screening, cytomegalovirus (CMV) status (donor/recipient) and CMV disease as determined by the CMV Drug Development Forum (17). Acute rejection (AR) and lymphocytic bronchiolitis (LB) were defined on histopathology as perivascular or peribronchiolar infiltrates as described by the International Society for Heart and Lung transplantation (ISHLT) guidelines (18). AR/LB was analyzed as a binary variable by comparing at least one AR/LB event during follow-up versus no single event of AR/LB. Severe grades of AR/LB (≥A2/B2) were analyzed separately. CLAD was defined consistent with the ISHLT guidelines as a persistent decline in forced expiratory volume in one second (FEV1) less than 80% of baseline (average of two best FEV1 values after LTx) in the absence of other confounding conditions (19). Graft loss was characterized as death or redo transplantation.

### Statistical Analysis

Results are presented in numbers (percentage) for binary variables or by the mean (± standard deviation) for continuous variables. The minor allele frequency (MAF) was calculated by dividing the number of times the minor T allele was observed by the total number of copies of all the alleles at the genetic locus of interest in the cohort. The chi-square test of independence was used to compare the count of T and G alleles between underlying diseases. The chi-square test of independence, Fisher exact test, *t*-test and Wilcoxon rank sum test were used where appropriate to compare clinical characteristics between patients without the MUC5B promoter polymorphism and patients who were homozygous or heterozygous for the MUC5B promoter polymorphism (GG vs. GT/TT).

Kaplan-Meier survival analysis was performed to compare graft loss and CLAD-free survival between patients without the MUC5B promoter polymorphism and patients who were homozygous or heterozygous for the MUC5B promoter polymorphism (GG vs. GT/TT). Observations were censored when the endpoint was not observed before October 17, 2019 and in the analysis of CLAD when the patient died without evidence of CLAD. Cox proportional-hazards model was used in multivariate analysis to investigate the association between rs35705950 (GG vs. GT/TT) and CLAD and graft loss, while controlling for age, gender, date of LTx, type of LTx in the ILD population and entire study cohort. Multivariate analysis by cox proportional-hazards model was additionally adjusted for the presence of at least one AR and LB event in the ILD population and for underlying disease in the entire study cohort. All variables were determined *a priori*.

To assess the influence of the MUC5B polymorphism (GG vs. GT/TT) on age at LTx, linear regression was used while adjusting for underlying ILD entity.

To investigate the association between MUC5B minor T allele (GG vs. GT/TT) and CMV disease, logistic regression was performed while adjusting for CMV status, age, gender, date of LTx and type of LTx.

No imputation of missing data was performed. All analyses were performed in R (version 4.0.3, R Project for Statistical Computing, Vienna, Austria). *p* < 0.05 was considered to represent statistical significance for all analyses.

## Results

### Minor Allele Frequency of MUC5B Polymorphism in LTx Cohort (1991–2015)

The study cohort encompassed 746 successfully genotyped patients: 159 (21.3%) patients had any form of ILD, 383 (51.3%) COPD/emphysema, 133 (17.8%) CF/BRECT, 68 (9.1%) PHT and 3 (0.4%) another diagnosis. The GG genotype was found in 601 patients (80.6%), the GT genotype in 132 patients (17.7%) and the TT genotype in 13 patients (1.7%). The MAF of the MUC5B variant was higher in ILD (17.6%) compared to COPD/emphysema (9.3%), CF/BRECT (7.5%), and PHT (7.4%) (*p* < 0.001) (Figure 1).

### Influence of MUC5B Polymorphism on CLAD/Graft Loss in LTx Cohort Transplanted for ILD (2004–2015)

To investigate the influence of the MUC5B promoter polymorphism on CLAD/graft loss, 117 ILD patients who were transplanted between 2004 and 2015 were included. The GG genotype was observed in 79 patients (67.5%), the GT genotype in 33 (28.2%) and TT genotype in 5 patients (4.3%). Among the 117 ILD patients, IPF was the most common subtype (29.9%; MAF of MUC5B promoter polymorphism 27.1%), followed by connective tissue disease associated-ILD (23.1%; MAF 22.2%), hypersensitivity pneumonitis (13.7%; MAF 12.5%), sarcoidosis (9.4%; MAF 9.1%), lymphangioleiomyomatosis (5.1%; MAF 8.3%), iNSIP (4.2%; MAF 20.0%) and exposure-induced ILD (4.2%; MAF 20.0%). Other diagnoses (10.3%; MAF 4.2%) included unclassifiable ILD, combined pulmonary fibrosis and emphysema, smoking-related ILD, histiocytosis X and eosinophilic pneumonitis. Native lung fibrosis phenotypes according to genotype and with corresponding MAF are shown in Supplementary Table S1. Patient characteristics stratified according to GG and GT + TT genotype are summarized in Table 1. The ILD subgroups did not significantly differ in year of LTx (*p* = 0.16), type of LTx (*p* = 0.20), immunosuppressive treatment (comparing azathioprine vs. mycophenolate mofetil: *p* = 0.59; and tacrolimus vs. cyclosporine: *p* = 0.08), CMV status (*p* = 0.52), preoperative use and dose of corticosteroids (*p* = 0.73 and *p* = 0.92), AR/LB history (*p* = 0.60 and *p* = 0.06) and AR/LB severe grades (≥A2: *p* = 0.61; ≥B2: *p* = 0.63). Patients with the MUC5B minor T allele were significantly older at time of transplantation (*p* = 0.01), more frequently male (*p* = 0.03) and had a shorter lung ischemic time (average time *p* = 0.01). More CMV infections were observed in patients with at least one minor allele (*p* = 0.02). To investigate the association between age at LTx and the MUC5B promoter polymorphism, linear regression adjusting for underlying entity was performed. Age at LTx was significantly associated with the underlying ILD entity, but not with the MUC5B polymorphism in multivariate analysis [coefficient: 2.83 95% CI (−0.61-6.28) *p* = 0.11]. To investigate the association between the MUC5B variant and CMV disease, logistic regression was performed adjusting for CMV status, age, gender, date of LTx and type of LTx. The MUC5B variant remained not significantly associated with CMV disease in this multivariate analysis [OR 1.15 95% CI (0.98–1.36) *p* = 0.09].

**TABLE 1:** Patient characteristics of the LTx cohort transplanted for ILD (2004–2015) according to genotype.

	GG (N = 79; 67.5%)	GT + TT (N = 38 (GT N = 33, TT N = 5); 32.5%)	*p*-value
Age at LTx (years)	50.9 ± 10.5	56.3 ± 7.9	0.008
Gender (female)	37 (46.8%)	9 (23.7%)	0.03
Date of LTx			0.16
2004–2007	24 (30.4%)	13 (34.2%)	
2008–2011	25 (31.6%)	17 (44.7%)	
2012–2015	30 (38.0%)	8 (21.1%)	
Type of LTx (SSL/HL vs. SL)	64-15 (81.0%–19.0%)	26-12 (68.4%–31.6%)	0.20
Treatment			
AZA-MMF—unknown	41-36-2 (51.9-45.6-2.5%)	23-15-0 (60.5-39.5-0.0%)	0.59
Tacrolimus-cyclosporine—unknown	49-28-2 (62.0-35.4-2.5%)	17-21-0 (44.7-55.3-0%)	0.08
CMV disease	15 (19.0%)	16 (42.1%)	0.02
CMV status			
D+/R +	8 (10.1%)	6 (15.8%)	0.52
D-/R-	31 (39.2%)	10 (26.3%)	
D+/R-	22 (27.8%)	10 (26.3%)	
D-/R+	15 (18.9%)	9 (23.7%)	
unknown	3 (3.8%)	3 (7.9%)	
Preoperative use of CS	54 (68.4%)	24 (63.2%)	0.73
Average dose of CS	6.6 ± 7.0	7.4 ± 9.4	0.92
Ischemic time (min)			
First lung	314 ± 112	271 ± 50	0.04
Second lung	482 ± 138	437 ± 86	0.15
Average	380 ± 125	323 ± 74	0.01
Acute rejection history			
Any AR	38 (48.1%)	21 (55.3%)	0.60
Severe AR (≥B2)	14 (17.7%)	9 (23.7%)	0.61
Any LB	24 (30.4%)	19 (50.0%)	0.06
Severe LB (≥B2)	17 (21.5%)	6 (15.8%)	0.63
CLAD	31 (39.2%)	19 (50.0%)	0.37
Graft loss	39 (49.4%)	21 (55.3%)	0.70

LTx, lung transplantation; SL, single lung transplantation; SSL, sequential single lung transplantation; HL, heart-lung transplantation; AZA, azathioprine; MMF, mycophenolate mofetil; CMV, cytomegalovirus; D, donor; R, receptor; CS, corticosteroids; AR, acute rejection; LB, lymphocytic bronchiolitis; CLAD, chronic lung allograft dysfunction.

**FIGURE 2:**
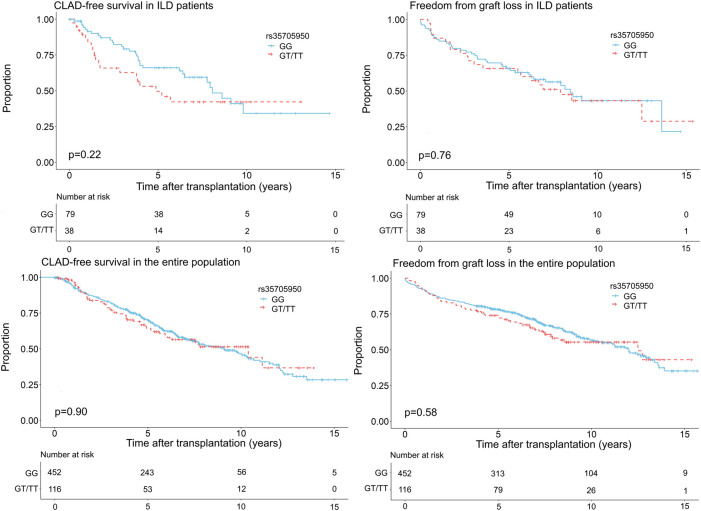
Freedom from chronic lung allograft dysfunction (CLAD) and graft loss according to genotype (GG vs. GT + TT) in the ILD population and the entire study cohort transplanted for a chronic end-stage respiratory disease between 2004 and 2015.

CLAD was diagnosed in 31 patients (39.2%) with the GG genotype and in 19 patients (50.0%) with the GT or TT genotype (*p* = 0.37). Graft loss was observed in 39 patients (49.4%) without a minor allele and in 21 patients (55.3%) with at least one minor allele (*p* = 0.70). Kaplan-Meier analysis showed no significant association between MUC5B promoter polymorphism and CLAD [HR 1.43 95% CI (0.81–2.44) *p* = 0.22] or between the MUC5B promoter polymorphism and graft loss [HR 1.09 95% CI (0.64–1.85) *p* = 0.76] in univariate analysis. Multivariate analysis by cox-proportional hazards model including the MUC5B variant, age at LTx, gender, date of LTx, type of LTx and presence of AR or LB, demonstrated no association between recipient MUC5B polymorphism and CLAD [HR 1.37 95% CI (0.70–2.68) *p* = 0.35], or between recipient MUC5B polymorphism and graft loss [HR 1.02 95% CI (0.55–1.89) *p* = 0.96] (Figure 2 and Table 2). Both multivariate models are presented in Supplementary Table S2.

**TABLE 2:** Univariate and multivariate analysis by cox-proportional hazards model of CLAD and graft loss in LTx cohort transplanted for ILD (2004–2015) and the entire LTx cohort transplanted for a chronic end-stage respiratory disease (2004–2015).

	HR [95% CI]	*p*-value
ILD population		
Univariate analysis		
CLAD	1.43 [0.81–2.44]	0.22
Graft loss	1.09 [0.64–1.85]	0.76
Multivariate analysis^a^		
CLAD	1.37 [0.70–2.68]^a^	0.35
Graft loss	1.02 [0.55–1.89]^a^	0.96
Total population		
Univariate analysis		
CLAD	1.02 [0.74–1.41]	0.90
Graft loss	1.10 [0.70–1.50]	0.58
Multivariate analysis^b^		
CLAD	0.96 [0.69–1.34]^b^	0.81
Graft loss	0.97 [0.70–1.35]^b^	0.87

a Adjusted for age at LTx, gender, date of LTx, type of LTx, and presence of AR, and LB.

b Adjusted for age at LTx, gender, date of LTx, type of LTx, and underlying disease.

CLAD, chronic lung allograft dysfunction; HR, hazard ratio; CI, confidence interval; ILD, interstitial lung disease.

### Influence of MUC5B Polymorphism on CLAD/Graft Loss in LTx Cohort Transplanted for a Chronic End-Stage Respiratory Disease (2004–2015)

To investigate the influence of the MUC5B promoter polymorphism on CLAD/graft loss, 568 patients transplanted for a chronic end-stage respiratory disease between 2004 and 2015 were included: 117 (20.6%) patients had any form of ILD, 307 (54.0%) COPD/emphysema, 105 (18.5%) CF/BRECT, 37 (6.5%) PHT and 2 (0.4%) another diagnosis. The GG genotype was found in 452 patients (79.6%), the GT genotype in 107 patients (18.8%) and the TT genotype in 9 patients (1.6%). Patient characteristics stratified according to GG and GT + TT genotype are summarized in Table 3. The subgroups differed significantly in age at LTx (*p* < 0.01), gender (*p* = 0.03), year of LTx (*p* = 0.01) and underlying disease (*p* = 0.01), but showed no difference in type of LTx (*p* = 0.05). CLAD and graft loss was observed in 193 patients (42.7%) and in 179 patients (39.6%) with a GG genotype compared to 46 patients (39.7%) and 49 patients (42.2%) with a GT or TT genotype (*p* = 0.63 and *p* = 0.68), respectively. No significant association between the MUC5B promoter polymorphism and CLAD [HR 1.02 95% CI (0.74–1.41) *p* = 0.90], or between the MUC5B promoter polymorphism and graft loss [HR 1.10 95% CI (0.70–1.50) *p* = 0.58] was observed in univariate analysis. In multivariate analysis including the MUC5B variant, age at LTx, gender, date of LTx, type of LTx and underlying disease, the MUC5B variant was neither associated with CLAD [HR 0.96 95% CI (0.69–1.34) *p* = 0.81] nor with graft loss [HR 0.97 95% CI (0.70–1.35) *p* = 0.87] (Table 2 and Figure 2). Both multivariate models are presented in Supplementary Table S3.

**TABLE 3:** Patient characteristics of LTx cohort transplanted for a chronic end-stage respiratory disease (2004–2015) according to genotype.

	GG [N = 452 (79.6%)]	GT + TT [(N = 116; GT N = 107; TT N = 9) (20.4%)]	*p*-value
Age at LTx (years)	49.4 ± 13.7	53.5 ± 11.6	0.004
Gender (female)	236 (52.2%)	47 (40.5%)	0.03
Type of LTx (SLL/HL vs. SL)	409 (90.5%)–43 (9.5%)	97 (83.6%)–19 (16.4%)	0.05
Date of LTx			0.007
2004–2007	139 (30.8%)	31 (26.7%)	
2008–2011	138 (30.5%)	53 (45.7%)	
2012–2015	175 (38.7%)	32 (27.6%)	
Indication for LTx			0.007
ILD	79 (17.5%)	38 (32.8%)	
COPD/emphysema	249 (55.1%)	58 (50.0%)	
CF/BRECT	90 (19.9%)	15 (13.0%)	
PHT	32 (7.1%)	5 (4.3%)	
Other diagnosis	2 (0.4%)	0 (0.0%)	
CLAD	193 (42.7%)	46 (39.7%)	0.63
Graft loss	179 (39.6%)	49 (42.2%)	0.68

LTx, lung transplantation; SL, single lung transplantation; SSL, sequential single lung transplantation; HL, heart-lung transplantation; ILD, interstitial lung disease; COPD, chronic obstructive pulmonary disease; CF, cystic fibrosis; BRECT, bronchiectasis; PHT, pulmonary hypertension; CLAD, chronic lung allograft dysfunction.

## Discussion

This is the first study reporting on the prevalence of the MUC5B minor T allele in chronic end-stage respiratory diseases and the association between this variant and post-transplant outcome. In the present study, MAF of the MUC5B promoter polymorphism was significantly higher in ILD than in other end-stage respiratory diseases. No association between recipient MUC5B polymorphism and post-transplant outcome was observed.

As expected, we found a higher prevalence of the MUC5B polymorphism in end-stage ILD with a MAF of 17.6%. This was lower than the previous reported MAF of 34–38% in IPF, 33% in RA-ILD, 24–32% in cHP and 29% in asbestosis, but in the present study, subgroups with a reported prevalence of the MUC5B variant equal to the normal population (i.e., sarcoidosis, SSc-ILD, myositis-associated ILD and antisynthetase syndrome) were also included (1–5,7–9). Indeed, our data on the prevalence of the MUC5B minor allele in the different ILD subgroups suggest a higher MAF in some ILD entities such as IPF (27.1%). The MUC5B promoter polymorphism was only associated with ILD as the MAF of the MUC5B polymorphism in the non-ILD end-stage respiratory diseases was comparable to the reported 9% in the normal Caucasian population (1). Although association between genetic variants and disease is not the same as causation, this finding suggests a specific role for the MUC5B promoter polymorphism and its associated increased mucin5b production in pulmonary fibrosis. How MUC5B exactly is involved in ILD susceptibility is still an unanswered question, but it highlights a potential role for the distal airways and mucus overproduction in the pathogenesis of pulmonary fibrosis (20,21).

The MUC5B minor T allele has been related to reduced mortality in IPF patients (10). Newton et al. confirmed this relationship in IPF patients but observed a worse transplant-free survival in interstitial pneumonia with autoimmune features (IPAF) patients and a trend toward worse transplant-free survival in CTD-ILD patients (22). Similarly, the presence of rs35705950 was of borderline statistical significance with worse survival in a cohort of cHP patients (2). Rare variants in telomere-related genes and short telomere lengths have been associated with progressive disease and worse survival across different ILD entities and with worse post-transplant outcome, even in the absence of notable syndromic clinical features of telomeropathies (2, 11–14, 22). Suggested mechanisms for this worse post-transplant outcome include defects in adaptive immunity and intolerance of the hematological stress of transplant-related myelosuppressive medications (12,14). For the MUC5B minor allele, no association with CLAD-free and graft survival was observed in both the ILD population and the total patient population. This could be explained by the fact that the MUC5B polymorphism only influences mucin5b producing cells, while telomere-related gene variants affect every organ system with increased cell turnover such as the bone marrow and immune system. Indeed, proposed mechanisms for reduced pre-transplant mortalities in IPF patients with a MUC5B minor T allele such as an enhanced host defense as a result of increased mucin production involve only the lung as organ system (23). After LTx, there is evidence for chimerism in a small percentage of epithelial cells in bronchial and alveolar tissue, but the majority of the cells originate from the donor (24). Regarding MAF of 9% in the normal Caucasian population, donor cells are more likely to express the major allele. It would therefore be very interesting to investigate the influence of donor MUC5B polymorphism on post-transplant outcome, but it was not possible to determine this within the scope of this study.

Although this is a very large cohort of lung transplant patients genotyped for rs35705950, there are several limitations to this study. First, this forms a single-center, retrospective cohort study and not all variables were present for each patient and genotyping could not be performed in all included patients. Second, the ILD population was relatively small and it was therefore not possible to draw conclusions about the MAF in the ILD subgroups or the influence of MUC5B polymorphism on post-transplant outcome in the specific ILD entities. Furthermore, while we investigated the influence of the recipient MUC5B polymorphism on post-transplant outcome, we were not able to do the same for donor MUC5B polymorphism. Lastly, some patients were only referred to our center for LTx and diagnostic work-up was performed in another center. The diagnostic certainty of the underlying lung disease in these cases is limited.

In conclusion, the prevalence of rs35705950 in chronic end-stage respiratory diseases in the context of COPD/emphysema, CF/BRECT and PHT was similar to the normal Caucasian population, while we confirmed the higher MAF in end-stage ILD. Therefore, the MUC5B promoter polymorphism is a very specific predictive factor for the presence of pulmonary fibrosis as it is only associated with pulmonary fibrosis and not with other chronic respiratory diseases. We found no association between recipient MUC5B polymorphism and post-transplant incidence of CLAD and graft loss in the ILD population and in the entire study cohort. While the MUC5B promoter variant is associated with better pre-transplant survival among IPF patients, recipient MUC5B promoter variant does not play a role in post-transplant outcome.

## Data Availability

The datasets presented in this article are not readily available because of ethical issues. Requests to access the datasets should be directed to the corresponding author.

## Abbreviations

AR, acute rejection; AZA, azathioprine; BRECT, bronchiectasis; CF, cystic fibrosis; cHP, chronic hypersensitivity pneumonitis; CI, confidence interval; CLAD, chronic lung allograft dysfunction; CMV, cytomegalovirus; COPD, chronic obstructive pulmonary disease; CS, corticosteroids; CTD-ILD, connective tissue disease-associated ILD; FEV1, forced expiratory volume in one second; HL, heart-lung transplantation; HR, hazard ratio; ILD, interstitial lung disease; iNSIP, idiopathic non-specific interstitial pneumonia; IPAF, interstitial pneumonia with autoimmune features; IPF, idiopathic pulmonary fibrosis; LTx, lung transplantation; LB, lymphocytic bronchiolitis; MAF, minor allele frequency; MCTD, mixed-connective tissue disease-associated ILD; MMF, mycophenolate mofetil; PHT, pulmonary hypertension; PM-ILD, polymyositis-associated ILD; RA-ILD, rheumatoid arthritis associated-interstitial lung disease; SL, single lung transplantation; SLE-ILD, systemic lupus erythematosus-associated ILD; SSc-ILD, systemic sclerosis associated-interstitial lung disease; SSL, sequential single lung transplantation.
