# Supplementary material for: The MUC5B Promoter Polymorphism is Not Associated With Non-ILD Chronic Respiratory Diseases or Post-transplant Outcome
Source: Transpl Int. 2022 May 16;35:10159. doi: 10.3389/ti.2022.10159 (PMC9149783; doi:10.3389/ti.2022.10159)
Supplement: Supplementary file 1 [file Table1.DOCX]

| Supplementary table 1: Native lung fibrosis phenotypes according to genotype and with corresponding minor allele frequency of ILD patients transplanted between 2004-2015 | | | | | |
| --- | --- | --- | --- | --- | --- |
|  | Total (N=117) | GG (N=79 67.5%) | GT (N=33, 28.2%) | TT (N=5, 4.3%)) | MAF |
| IPF | 35 (29.9%) | 18 (51.4%) | 15 (42.9%) | 2 (5.7%) | 27.1% |
| CTD-ILD   - PM-ILD - SSc-ILD - SLE-ILD - RA-ILD - MCTD-ILD | 27 (23.1%)  5 (18.5%)  8 (29.6%)  3 (11.1%)  10 (37.0%)  1 (3.7%) | 18 (66.7%)  4 (70.0%)  7 (87.5%)  0 (0.0%)  6 (60.0%)  1 (100.0%) | 6 (22.2%)  1 (20.0%)  1 (12.5%)  2 (66.7%)  2 (20.0%)  0 (0.0%) | 3 (11.1%)  0 (0.0%)  0 (0.0%)  1 (33.3%)  2 (20.0%)  0 (0.0%) | 22.2%  10.0%  6.3%  66.6%  30.0%  0.0% |
| Hypersensitivity pneumonitis | 16 (13.7%) | 12 (75.0%) | 4 (25.0%) | 0 (0.0%) | 12.5% |
| Sarcoidosis | 11 (9.4%) | 9 (81.8%) | 2 (18.2%) | 0 (0.0%) | 9.1% |
| Lymphangioleiomyomatosis | 6 (5.1%) | 5 (83.3%) | 1 (16.7%) | 0 (0.0%) | 8.3% |
| iNSIP | 5 (4.2%) | 3 (60.0%) | 2 (20.0%) | 0 (0.0%) | 20.0% |
| Exposure-induced ILD | 5 (4.2%) | 3 (60.0%) | 2 (20.0%) | 0 (0.0%) | 20.0% |
| Other diagnoses | 12 (10.3%) | 11 (91.7%) | 1 (8.3%) | 0 (0.0%) | 4.2% |

MAF: minor allele frequency; IPF: idiopathic pulmonary fibrosis; CTD-ILD: connective tissue disease-associated ILD; PM-ILD: polymyositis-associated ILD; SLE-ILD: systemic lupus erythematosus-associated ILD; RA-ILD: rheumatoid arthritis-associated ILD; MCTD: mixed-connective tissue disease-associated ILD; iNSIP: idiopathic non-specific interstitial pneumonia
