# Supplementary material for: The MUC5B Promoter Polymorphism is Not Associated With Non-ILD Chronic Respiratory Diseases or Post-transplant Outcome
Source: Transpl Int. 2022 May 16;35:10159. doi: 10.3389/ti.2022.10159 (PMC9149783; doi:10.3389/ti.2022.10159)
Supplement: Supplementary file 2 [file Table2.docx]

| Supplementary table 2: multivariate analysis by cox-proportional hazards model of CLAD and graft loss in LTx cohort transplanted for ILD (2004-2015) | | | | |
| --- | --- | --- | --- | --- |
|  | **HR 95%CI** | **p-value** | **HR 95%CI** | **p-value** |
| rs35705950  Age at LTx  Gender (female)  Date of LTx  2004-2007  2008-2011  2012-2015  Type of LTx (SSL/HL)  Any AR  Any LB | **CLAD**  1.37 [0.70-2.68]  1.01 [0.96-1.05]  1.48 [0.75-2.91]  NA  0.66 [0.34-1.29]  0.32 [0.12-0.85]  0.71 [0.29-1.71]  1.44 [0.79-2.61]  0.81 [0.42-1.57] | 0.35  0.74  0.26  NA  0.22  0.02  0.45  0.23  0.53 | **Graft loss**  1.02 [0.55-1.89]  1.03 [0.99-1.07]  1.01 [0.55-1.89]  NA  0.57 [0.31-1.07]  0.58 [0.25-1.34]  0.59 [0.27-1.26]  0.81 [0.47-1.38]  0.63 [0.35-1.16] | 0.96  0.20  0.98  NA  0.08  0.21  0.17  0.43  0.14 |

HR: hazard ratio; CI: confidence interval; LTx: lung transplantation; SSL: sequential single lung transplantation; HL: heart-lung transplantation; AR: acute rejection; LB: lymphocytic bronchiolitis; CLAD: chronic lung allograft dysfunction
