# Supplementary material for: The MUC5B Promoter Polymorphism is Not Associated With Non-ILD Chronic Respiratory Diseases or Post-transplant Outcome
Source: Transpl Int. 2022 May 16;35:10159. doi: 10.3389/ti.2022.10159 (PMC9149783; doi:10.3389/ti.2022.10159)
Supplement: Supplementary file 3 [file Table3.docx]

| Supplementary table 3: multivariate analysis by cox-proportional hazards model of CLAD and graft loss in LTx cohort transplanted for a chronic end-stage respiratory disease (2004-2015) | | | | |
| --- | --- | --- | --- | --- |
|  | **HR 95%CI** | **p-value** | **HR 95%CI** | **p-value** |
| rs35705950  Age at LTx  Gender (female)  Date of LTx  2004-2007  2008-2011  2012-2015  Type of LTx (SSL/HL)  Underlying disease  ILD  COPD/emphysema  CF/BRECT  PHT | **CLAD**  0.96 [0.69-1.34]  1.01 [0.99-1.02]  1.25 [0.97-1.63]  NA  0.92 [0.67-1.27]  0.72 [0.49-1.05]  0.79 [0.52-1.21]  NA  1.02 [0.71-1.44]  0.56 [0.32-0.97]  0.76 [0.40-1.45] | 0.81  0.64  0.09  NA  0.62  0.09  0.28  NA  0.93  0.04  0.41 | **Graft loss**  0.97 [0.70-1.35]  1.01 [1.00-1.03]  0.97 [0.74-1.27]  NA  0.66 [0.48-0.91]  0.71 [0.48-1.05]  0.56 [0.39-0.81]  NA  0.68 [0.49-0.94]  0.48 [0.26-0.86]  1.00 [0.56-1.78] | 0.87  0.13  0.83  NA  0.01  0.08  <0.01  NA  0.02  0.01  0.99 |

HR: hazard ratio; CI: confidence interval; LTx: lung transplantation; SSL: sequential single lung transplantation; HL: heart-lung transplantation; CLAD: chronic lung allograft dysfunction; ILD: interstitial lung disease; COPD: chronic obstructive pulmonary disease; CF: cystic fibrosis; BRECT: bronchiectasis; PHT: pulmonary hypertension
